# Smart Wearables with Sensor Fusion for Fall Detection in Firefighting

**DOI:** 10.3390/s21206770

**Published:** 2021-10-12

**Authors:** Xiaoqing Chai, Renjie Wu, Matthew Pike, Hangchao Jin, Wan-Young Chung, Boon-Giin Lee

**Affiliations:** 1 School of Computer Science, Faculty of Science and Engineering, The University of Nottingham Ningbo China, Ningbo 315100, China; xiaoqing.chai@nottingham.edu.cn (X.C.); renjie.wu@nottingham.edu.cn (R.W.); matthew.pike@nottingham.edu.cn (M.P.); 2 Ningbo Municipal Public Security Fire Brigade, Haishu Detachment, Ningbo 315100, China; jhc_2006@163.com; 3 Department of Electronic Engineering, Pukyong National University, Busan 48513, Korea; wychung@pknu.ac.kr

**Keywords:** fall detection system, deep learning, wearable IOT technology, inertial measurement unit (IMU), multisensory fusion

## Abstract

During the past decade, falling has been one of the top three causes of death amongst firefighters in China. Even though there are many studies on fall-detection systems (FDSs), the majority use a single motion sensor. Furthermore, few existing studies have considered the impact sensor placement and positioning have on fall-detection performance; most are targeted toward fall detection of the elderly. Unfortunately, floor cracks and unstable building structures in the fireground increase the difficulty of detecting the fall of a firefighter. In particular, the movement activities of firefighters are more varied; hence, distinguishing fall-like activities from actual falls is a significant challenge. This study proposed a smart wearable FDS for firefighter fall detection by integrating motion sensors into the firefighter’s personal protective clothing on the chest, elbows, wrists, thighs, and ankles. The firefighter’s fall activities are detected by the proposed multisensory recurrent neural network, and the performances of different combinations of inertial measurement units (IMUs) on different body parts were also investigated. The results indicated that the sensor fusion of IMUs from all five proposed body parts achieved performances of 94.10%, 92.25%, and 94.59% in accuracy, sensitivity, and specificity, respectively.

## 1. Introduction

**Figure 1:**
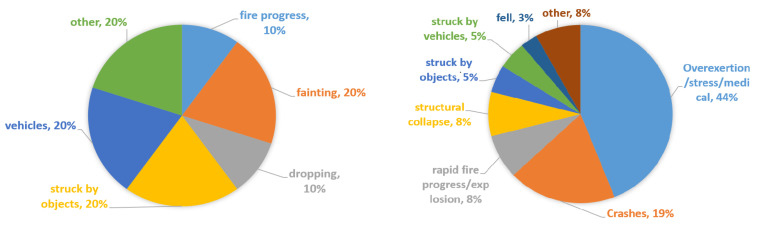
Comparison of the causes of firefighters’ fatalities in China (**left**) and in the USA (**right**), 2018 [1,2].

Falling activities, including struck, dropping, and fainting, are some of the primary causes of firefighter fatalities in China (Figure 1) [1]. In the United States in 2018, overexertion and stress were the leading causes of death in firefighters, according to a US National Fire Protection Association (NFPA) report [2]. A major cause of this difference is that the personal protective equipment (PPE) for fall detection still requires improvement to better guarantee safety [3].

**Figure 2:**
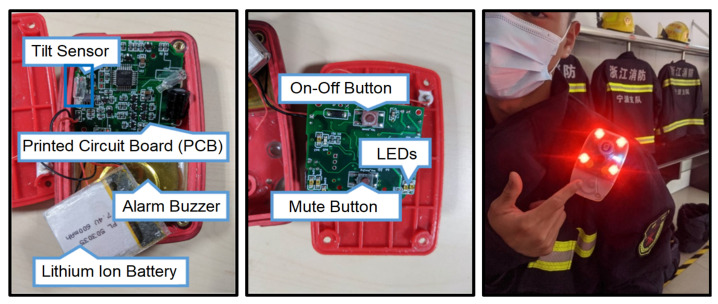
PASS device equipped by a firefighter in China.

The current FDS used by firefighters in China is a standard personal-alert safety system (PASS) device for detecting a firefighter’s immobility (see Figure 2). It produces a high-volume sound if no motion is detected after a short period (typically 30 s). There are concerns, however, that this delay may be a critical deciding factor between life and death in a real emergency.

In addition, the lack of training and experience is also a major cause. According to the 2019 annual report from the International Association of Fire and Rescue Services (CTIF) [4], among a total of 7,630,000 firefighters in China, only 130,000 are career firefighters, accounting for only 1.7%. The rest are all volunteer firefighters. However, the percentage of career firefighters is approximately 33.2% in the USA. These young volunteer firefighters with short training periods and little firefighting experience are often unable to master complex firefighting skills, especially in dangerous situations, such as burning floors or unstable building structures [3].

In the majority of recorded fatalities on the fireground, the incident commander was unaware of a fallen firefighter and, therefore, unable to instigate a rescue in time. The smoke-filled environment also decreased firefighters’ ability to identify a peer’s injuries and safety in a timely manner. In general, the firefighters’ education principle in China was to learn and gain experience through actual firefighting tasks, with a high learning cost of their life and safety [5]. These issues highlight the need for smart protection measurements to detect firefighters’ falling activities to ensure their safety during firefighting missions.

Several studies [6,7,8] investigated the application of context-aware systems (CASs) and wearable sensors for FDSs. A CAS generally integrates a set of vision systems with cameras and other sensors, such as microphones or vibration sensors, placed in a well-lit environment to monitor the user within the range of view [9]. Wearable sensors are commonly used to analyze a human’s fall activities, based on the motion pattern [10] and physiological status [11]. Even though FDSs have been widely deployed in the healthcare sector, especially for elderly people, the system is targeted at detecting slow-falling movements, which is not suitable for firefighters.

The current PASS device has the following deficiencies: (1) a long delay of approximately 30 s to raise the alert; (2) audible alarms are insufficient, if a firefighter is far away from his peers or in a noisy environment; and (3) the action commander cannot receive the alert soon enough to carry out a timely rescue. This study aims to develop a smart FDS, integrated with wearable sensors, for detecting the fall activities of firefighters, especially in harsh environments, and alert the action commander in time to organize a rescue. The proposed FDS applied GA10 & CCC certified national personal-protective clothing (PPC) with embedded motion sensors to gather firefighter moving-activity data. The key innovative aspects of this study are as follows.

- Performance evaluation of firefighter fall detection based on motion sensors that are placed in different parts of the body (PPC), including chest, elbows, wrists, thighs, and ankles.
- Aim to build a high realistic falling related movements dataset through collaboration with real firefighters.
- Proposes a novel fall-detection model which is trained with deep learning approach that can classify actual falls and fall-like events.

The rest of the paper is organized as follows: Section 2 presents a literature review of various FDS approaches, detailing the advantages and disadvantages; Section 3 describes the proposed smart wearable PPC prototype design which is used in for data collection, and discusses the details of the proposed FDS algorithms; Section 4 presents the evaluation of FDS models with various combinations of IMUs, followed by the discussion of the fall detection performance in each activity, and subsequently perform results comparison with the existing works; Finally, we conclude with a discussion of our future work in Section 5.

## 2. Related Works

Fall-detection methods can be categorized into two approaches: vision-based and non-vision-based. In a vision-based approach, fall detection is modeled based on images or videos obtained from different types of cameras [9]. Iazzi et al. [12] proposed a vision-based fall detector by isolating the different activities using a simple threshold method. The approach first applied background subtraction to images obtained from an RGB camera to compute a human silhouette. It then classified the activities, such as lying, bending, sitting, and standing, by comparing the percentage of a human silhouette on the ground with a predefined threshold. However, they also indicated that the system was inadequate if multiple people were in the vision view. Moreover, a study by [13] presented an active vision system for fall detection, based on skeleton movement captured by a 3D camera. Several works [14,15] also analyzed head-moving patterns, where large head movements indicated a high possibility of falling.

Nevertheless, vision-based solutions have several intractable issues in firefighting applications, such as poor performance in dark, low-light, or smoke-filled environments, limited range of vision, and views obstructed by obstacles in the field [16]. Hence, some studies have explored the potential use of non-vision-based approaches to reduce the constraints of vision-based approaches. The advantages of such approaches include lower costs, in terms of algorithm computation and image processing, privacy protection, portability, and less affected by the environment [17].

IMUs embedded in mobile devices, such as smartphones and smartwatches, are commonly used by many researchers for fall detection, typically in healthcare areas for the elderly [18,19,20,21,22,23]. These studies extract motion data from a common nine degrees-of-freedom (DOF) IMU for classifying fall activities, assuming that the smartphone is stored in the pants pocket or a smartwatch is worn on the wrist. Nonetheless, carrying a smartphone into a fireground can affect the performance of firefighting activities and, thus, is prohibited. Hence, this approach is inapplicable and not considered in this study. Instead, a sensor-fusion technique that integrates diverse wearable sensors for fall detection is a better alternative.

Lee et al. [24] added an IMU with an RGB camera to improve the fall-recognition rate and reduce the false-detection rate of falls. The study utilized a robot equipped with a camera that could move toward the fall subject for further verification of a fall, if the IMU-based classifier first predicted a fall-like activity. On the other hand, Kwolek et al. [25] integrated a Kinect sensor and an IMU for fall detection, reducing the false fall alerts, which improved the overall fall-detection performance from 90% (using only depth information from the Kinect sensor) to 98.33%.

Sensor-fusion techniques with integrated vision-based sensors are not useful for improving the fall detection of firefighters in smoke-filled and harsh environments. In addition, Chen et al. [26] developed a shoe integrated with a barometer and IMU for a stair-based fall-risk detection system by measuring foot movements going upstairs or downstairs. The study also revealed that the multisensory system performed better than a single-sensor system in falling-risk evaluations.

Several studies have emphasized the use of PASS and PPC in fall detection for firefighters [27,28,29,30]. Van Thanh et al. [27] proposed a device wearable on the waist, integrated with a triaxial accelerometer and a carbon monoxide (CO) sensor to monitor falls in fire environments. The CO sensor is specifically utilized for detecting falls caused by broken air-support devices. They improved their system in [28] with a sensor-fusion approach using a 9-DOF IMU, a CO sensor, and a barometer. However, the datasets they collected are not publicly available.

Geng et al. [29] proposed a novel health-monitoring system with electrocardiogram (ECG), electroencephalogram (EEG), and blood-pressure measurements to recognize the motion events of firefighters by extracting features obtained from the on-body radio-frequency (RF) channel. The average true classification rate is 88.69%.

Moreover, Blecha et al. [30] proposed functional wearable PPC for firefighters that could monitor their physiological status (heart rate and temperature), detect firefighter movements, and measure environmental information, such as the relative humidity and concentration of toxic gases. In addition, they also designed a commander control unit as a terminal to receive functional data from the PPC and alert the commander if any safety risk to the firefighters was detected.

In summary, among existing fall-detection studies using wearable-type sensors, most studies are targeted at detecting the falls of the elderly. Moreover, few to no public datasets are available for the study and analysis of firefighter fall detection. Our work makes important contributions that compensate for these shortcomings.

(1) Building a dataset by collecting motion data of actual firefighters, including falls and fall-like activities, for academic research purposes,
(2) Investigating the optimization of motion sensors in fall-activity classification, in terms of their quantity and placement on firefighter protective clothing, and
(3) Presenting a fall-detection framework applied for firefighters, especially when they are often working in high-stress situations.

## 3. Materials and Methods

This section discusses using the proposed novel design of smart PPC for firefighters to gather firefighter motion data. Next, the experimental setup for data collection is discussed, followed by a detailed description of the proposed FDS.

### 3.1. Smart PPC Prototype

**Figure 3:**
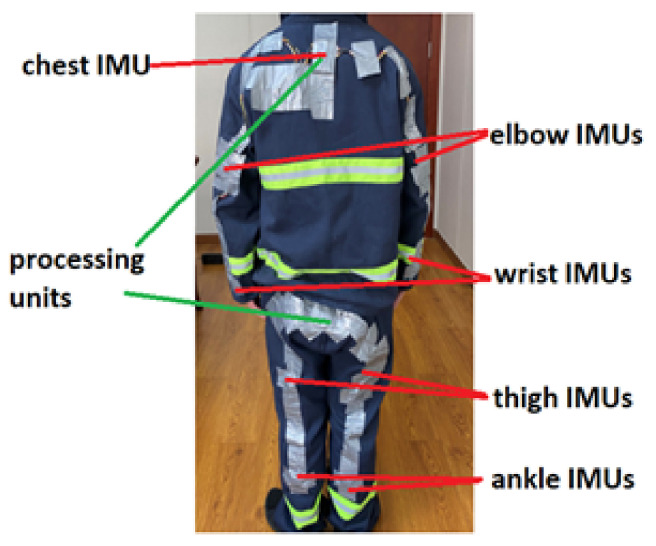
Placement of motion sensors that are mapped to the body parts where the motion data are critical for fall-detection computation.

The placement of wearable sensors plays an essential role in recognizing falls with a high accuracy rate [10]. The important motion data that contribute to fall-event detection are associated with the moving patterns of the chest, elbow, and wrist from the upper body, and the thigh and ankle from the lower part of the body. Hence, BNO055 IMUs [31], consisting of a triaxial accelerometer, triaxial gyroscope, and triaxial magnetometer, are integrated with wired connections on the back of the protective jacket (PJ) and protective trousers (PT), as shown in Figure 3. The angular velocity ranges from ±125 deg/s to ±2000 deg/s with a low-pass filter bandwidth from 523 Hz to 12 Hz, while the acceleration ranges from ±2 g to ±16 g with a low-pass filter bandwidth from 1 kHz to 8 Hz, and the measurement range of the magnetometer is about ±4800 uT with a resolution of 0.3 uT. The maximum output rate of 9-DOF fusion data is 100 Hz. However, considering fast data transmission can result in low data receiving efficiency, both sampling rate of the 9-DOF data and wireless transmission rate were set to 15 Hz in this study after several trials for optimization. An IMU was not placed on the shoulder because shoulder movement is always associated with chest movement.

**Figure 4:**
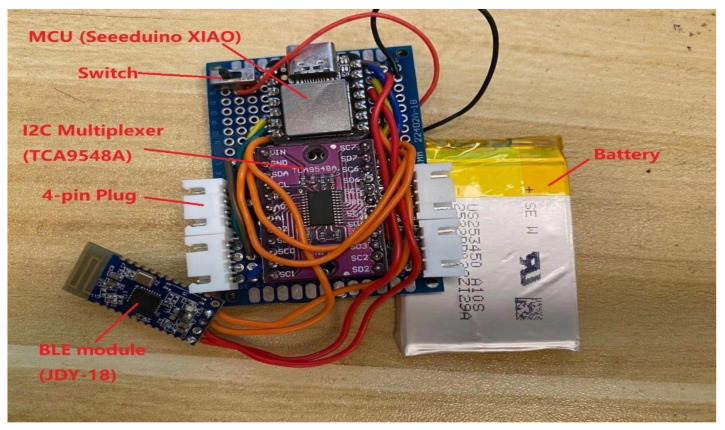
Processing unit that consists of an MCU, an I2C multiplexer, a BLE 4.2 module, and a lithium-ion battery.

Two processing units are placed on the chest and waist for receiving and transmitting IMU data from the PJ and PT, respectively. Each processing unit consists of a Seeeduino XIAO micro-controller unit (MCU) with a 20 × 17.5 mm${}^{2}$ size and 3.3-V power consumption [32], a TCA9548A 1-to-8 I2C multiplexer [33] for multisensor connections, a Bluetooth low-energy (BLE) 4.2 module [34], and a 3.7-V 400-mAh lithium-ion battery [35], as illustrated in Figure 4. The IMU sensors are connected to a processing unit with wires soldered onto the PJ and PT.

**Figure 5:**
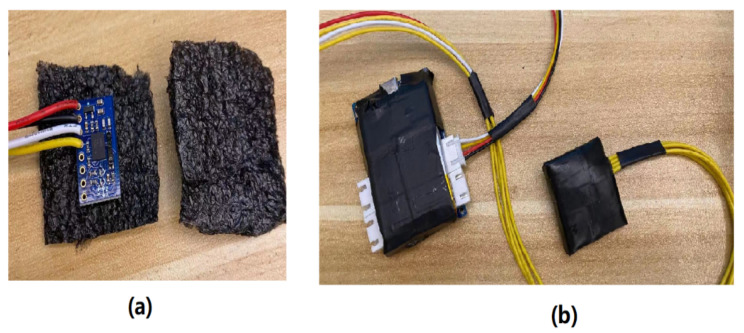
(**a**) Foam board is placed on the IMU for component protection and (**b**) rubber tape is used to secure the wire connections between the processing unit and IMUs.

**Figure 6:**
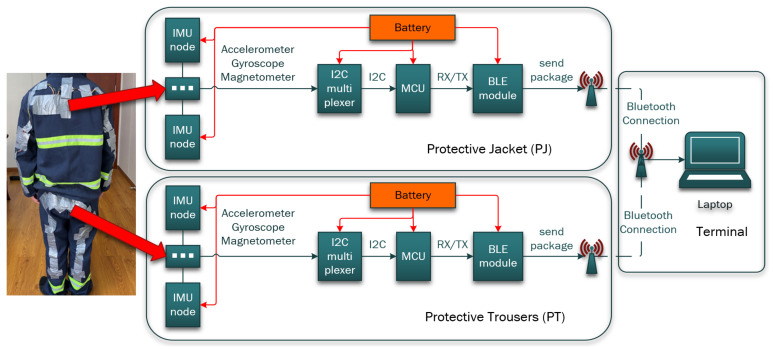
Overall design of the communication framework from the PJ and PT to a terminal via BLE 4.2 wireless transmission.

Table 1 summarizes the components of the sensing module with their respective specifications. To reduce the risk of destroying the components (including IMUs, processing units, and wires) during the data collection, foam boards were placed on top of all the components for protection, and rubber tape was used to secure the wire connections between the processing unit and IMUs, as depicted in Figure 5. Finally, the IMU data from the PJ and PT are transmitted to a terminal via BLE 4.2 for further processing, as shown in Figure 6.

### 3.2. Dataset Collection

Yan et al. [36] states that falls may be categorized into four distinct types: basic forward, backward, left, and right lateral falls. The fall-like activities can be further subdivided into basic forward, backward, left, and right lateral falls. However, a fall event is much more complicated during firefighting activities. Some specific activities, such as slipping, sliding, and fainting, can also result in falls [37]. Several existing studies [36,38,39] demonstrate the challenges of differentiating fall-like activities, such as sitting quickly, jumping onto a bed, and lying down slowly, from an actual fall. However, these existing fall datasets are not suitable for detecting the falls of firefighters because most of the recorded activities do not closely simulate realistic falling events in a fireground, such as jumping onto a bed.

This study initiated a collaboration with firefighters from the Haishu District Fire Brigade from Ningbo City, Zhejiang Province, China, to obtain realistic fall events by firefighters, based on their experiences. Fourteen male firefighters (with one to three years of firefighting experience, ages between 21 and 24 years old, with heights between 1.7 and 1.88 m) voluntarily participated in the data collection. Six of them were career firefighters and the other eight were volunteer firefighters.

**Figure 7:**
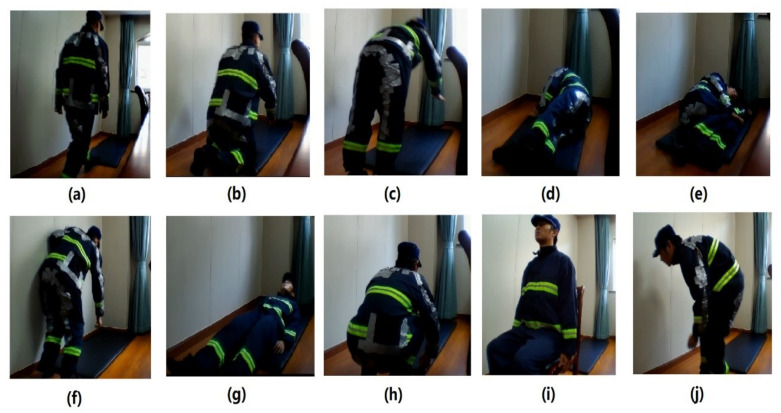
Demonstration of a firefighter with the proposed PJ and PT performing different types of falls and fall-like activities, including (**a**) walking to a mat before falling, (**b**) forward fall with the knees, (**c**) forward fall with the hands, (**d**) left side of an inclined fall, (**e**) right side of an inclined fall, (**f**) slow forward fall with a crouch first, (**g**) backward fall, (**h**) fall-like crouching, (**i**) fall-like sitting, and (**j**) fall-like walking with a stoop.

Six types of fall activities were collected, including a forward fall with the knees, forward fall with the hands, left and right sides of inclined falls, backward fall, and a slow forward fall with a crouch. Three other activities, including crouching, sitting, and walking with a stoop, were also collected as fall-like activities. Each firefighter was requested to put on the developed PJ and PT and simulate falls and fall-like activities, based on their firefighting experience. The details of the falls and fall-like activities are illustrated in Figure 7. The number of trials for each activity and total trials are summarized in Table 2.

**Figure 8:**
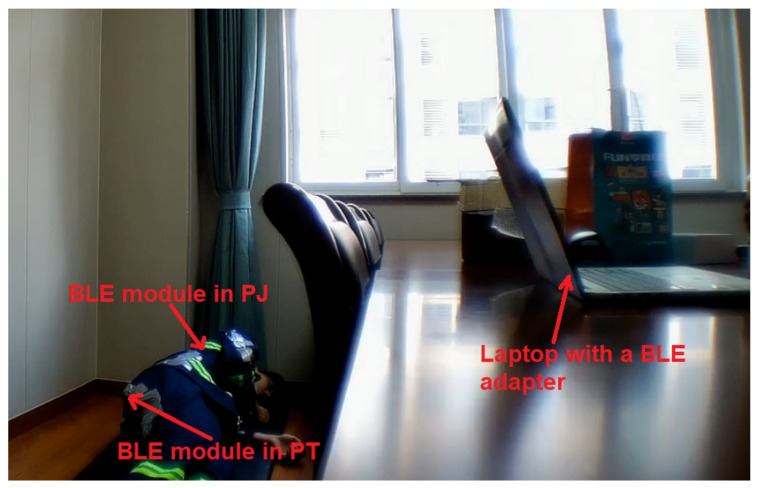
Data collection, via BLE 4.2 wireless transmission to a laptop, of IMU data from the PJ and PT.

According to Figure 8, IMU data from the PJ and PT are transmitted to a laptop via BLE 4.2 wireless communication with a 15-Hz sampling rate. A fall action consists of three phases: early fall, impact, and recovery [40]. As the aim of this study is to target the falling event, the recorded data of the early falling phase are labeled as falls, whereas the rest are labeled as non-falls.

### 3.3. Framework

**Figure 9:**
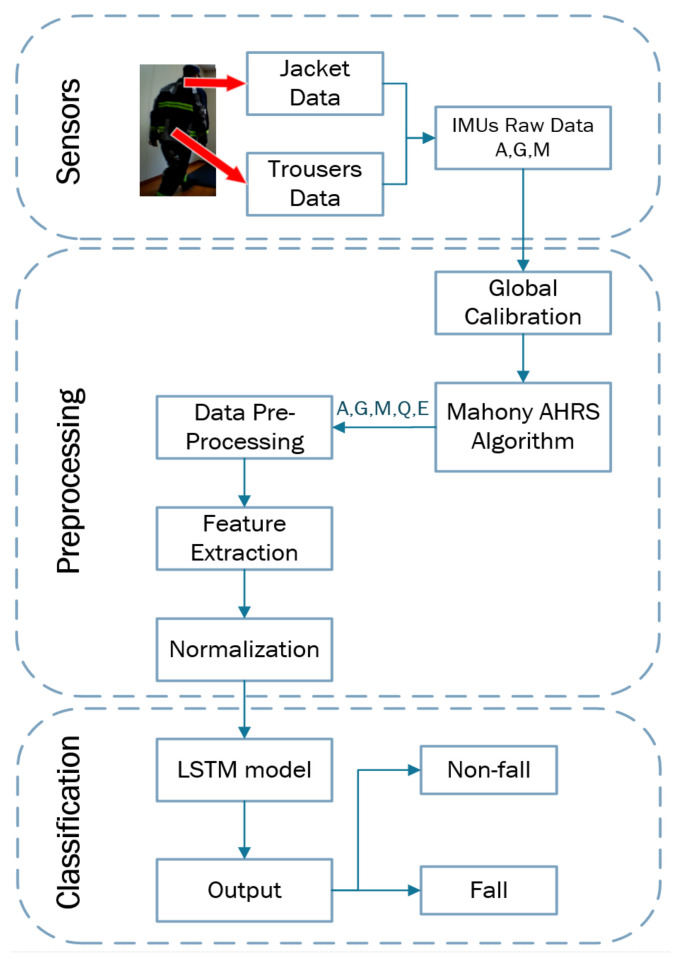
Flowchart of the FDS algorithm, where A = triaxial accelerometer data, G = triaxial gyroscope data, M = triaxial magnetometer data, Q = quaternion data, and E = Euler angles.

The proposed wearable FDS is composed of three modules: (1) sensing, (2) pre-processing, and (3) classification, as illustrated in Figure 9.

#### 3.3.1. Global Calibration of IMUs

**Figure 10:**
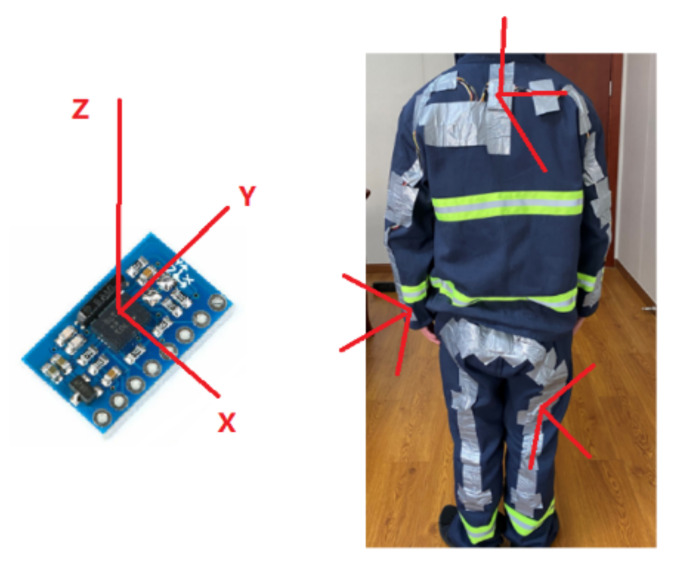
Local IMU coordinate system on PJ and PT.

Each IMU has a tri-axial coordinate system, as depicted in Figure 10, which can be used to determine the position and orientation of an object. In fact, an FDS with multiple IMUs placed on different parts of the PPC needs further calibration to synchronize and unify the coordinate system as a single entity. To achieve a global calibration for IMUs on the PJ and PT, two common reference vectors are required, as proposed by O’Donovan et al. [37].

**Figure 11:**
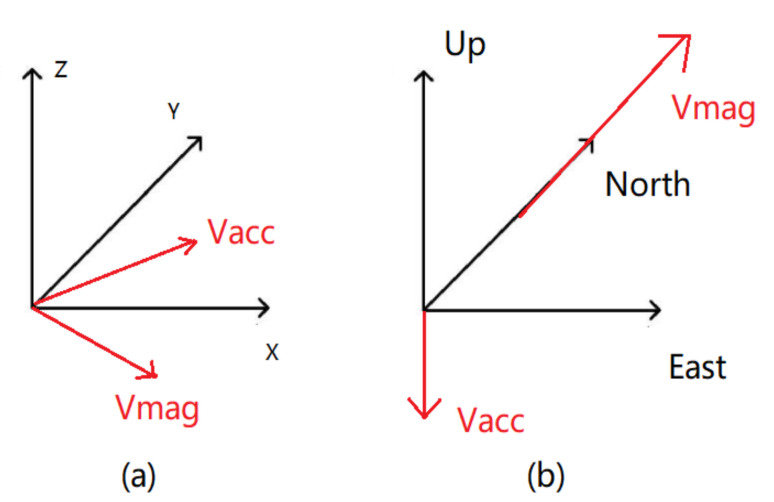
Global calibration. (**a**) Local coordinate system and vectors of Vacc and Vmag and (**b**) NEU coordinate system and vectors of Vacc and Vmag.

The magnetic field vector (Vmag), which points to the north, is selected as one of the common reference vectors, assuming no magnetic interference and that the magnetic field in the vicinity of each IMU is the same. The other reference vector is the acceleration vector (Vacc) in a quasi-static condition; it can be regarded as the gravity vector pointing to the ground. The IMU local coordinates can thus be rotated to the north-east-up (NEU) earth coordinate system (right-hand rule). Figure 11 presents the local and global coordinate systems with two common reference vectors Vmag and Vacc. The calibration works in the way that the subject should stand still initially, and it will take 1 second (15 samples) to compute the average values of Vacc and Vmag. The main purpose of the calibration is to convert the local coordinate system of each IMU to the unified NEU system. With the values of Vacc and Vmag as the reference vectors, the rotation matrix of these two coordinate systems can be computed, and hence, update the collected raw data of each IMU referred to the NEU system.

#### 3.3.2. Data Pre-Processing

The Mahony attitude and heading reference system (AHRS) [41] is utilized to compute the quaternion and Euler angles from the raw data received from the terminal. The rotation matrix of each IMU node can be derived as follows:

(1) q=w+xi+yj+zk

(2) $$R\left(q\right)=\left[\begin{array}{ccc} 2w^{2}+2x^{2}-1 & 2xy-2zw & 2xz+2yw\\ 2xy+2zw & 2w^{2}+2y^{2}-1 & 2yz-2xw\\ 2xz-2yw & 2yz+2xw & 2w^{2}+2z^{2}-1 \end{array}\right],$$

where *q*, *w*, *x*, *y*, and *z* represent a quaternion value that consists of a real number (*w*) and imaginary values on three imaginary axes (*i*, *j*, *k*).

Each IMU delivered 13 outputs, including tri-axial acceleration (m/s${}^{2}$), tri-axial angular rate (deg/s${}^{2}$), four-point quaternion data, and the derived roll, pitch, and yaw angles. To represent the variation of the different movements of a firefighter, given a defined period, features including the mean (μ) (Equation (3)), range (*R*) (Equation (4)), standard deviation (σ) (Equation (5)), and mean absolute deviation (MAD) (Equation (6)), are extracted from the 13 outputs, and calculated using 0.5 s of data each, with a window size of 0.1 s. As a result, each IMU generates 52 vectorized features (13 outputs × 4 arithmetic calculations), in which the total number of input features to the classifier is 468 features (52 features × 9 IMUs).

(3) $$\mu =\frac{1}{N}\Sigma x\left[k\right]$$

(4) R=maxx−minx

(5) $$\sigma =\sqrt{\frac{1}{N}\Sigma {\left(x\left[k\right]-\mu \right)}^{2}}$$

(6) $$MAD=\frac{1}{N}\Sigma x\left[k\right]-\mu$$

#### 3.3.3. Recurrent Neural Network Classifier

**Figure 12:**
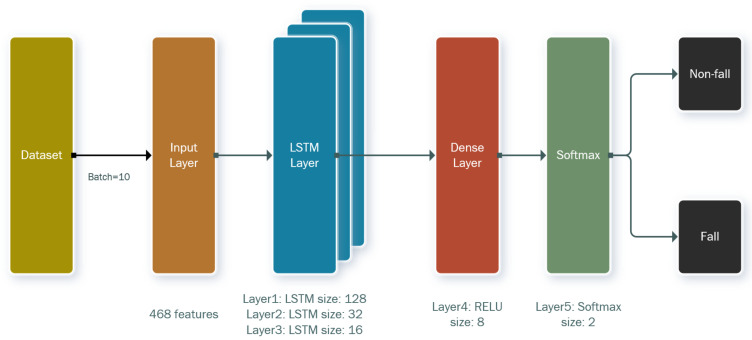
LSTM network architecture.

Figure 12 illustrates the neural network architecture, which includes three long short-term memory (LSTM) layers with 128 units, 32 units, and 16 units, respectively, one dense layer with an eight-unit rectified linear unit (ReLU) activation function (Equation (7)), and a softmax activation function with two units (Equation (8)). This represents the probability of non-fall and fall activities using one-hot encoding. The adaptive moment estimation (Adam) algorithm with a learning rate of 0.01 is applied as the optimizer of the model, and the sparse categorical cross-entropy algorithm, shown in Equation (9), is used as the loss function in the model. The batch size is set to 10, which represents the user’s action in one second. The dataset is divided into 80% for training and 20% for testing.

(7) ReLU=max(0,x)

(8) $$p\left(y_{i}\right)=\frac{e^{y_{i}}}{{\Sigma^{n}}_{j=1}e^{y_{i}}}$$

(9) $$loss\left(y_{i}\right)=-\log p\left(y_{i}\right)$$

## 4. Results

**Figure 13:**
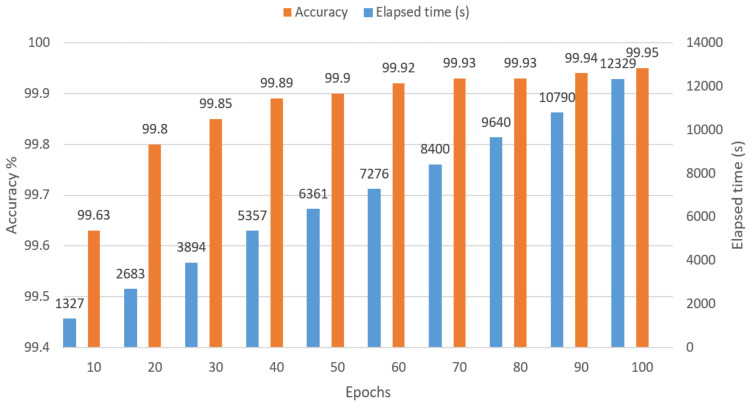
Training results of the LSTM model using different epochs.

First, the performance of the trained model with the proposed LSTM architecture is presented in Figure 13 for every 10 epochs. It uses an RTX2060 6G RAM GPU with an Intel i7-9700 CPU (3.0 GHz). The results indicate a slow increment in the accuracy rate after 40 training epochs, with the highest accuracy of 99.95% obtained after 100 training epochs, using all the sensor data.

Meanwhile, this study has allocated 30 different combinations of sensor’s placements to further investigate the optimization of the sensors’ placements and the quantity of sensors allocated for the fall detection of firefighters. Five positions were coded, representing the placement of the IMU on the protective clothing, as listed in Table 3, including the chest, elbows, wrists, thighs, and ankles. The performance of each combination’s model is evaluated, including the trained results using accuracy and loss, and the overall test performance using the following widely used metrics:

- **AUC:** Area under the receiver operating characteristic (ROC) curve.
- **Specificity (Sp):** the ability to predict negative samples.
- **Sensitivity (Se):** also called recall; the ratio means the accuracy among all predictions of falling.
- **Accuracy (Ac):** the accuracy among all predictions, both positive and negative.

The equations for specificity, sensitivity, and accuracy are shown in Equations (10)–(12), respectively.

(10) $$SE=\frac{TP}{TP+FN}$$

(11) $$Sp=\frac{TN}{TN+FP}$$

(12) $$Ac=\frac{TP+TN}{TP+TN+FP+FN}$$

Table 4 illustrates the performance of these 30 models. The training accuracy of the models after 100 epochs is nearly identical and most reach over 99.90%. In general, the best fall-detection performance improved gradually with the addition of more IMUs placed on different sections of the cloth, with the highest Ac, Se, and Sp achieved at 94.10%, 92.25%, and 94.59%, respectively, for all IMUs included as proposed.

It is important to note that the IMU placed on the chest plays an important role in detecting the fall of the firefighter, as the combination of EWTA, where the chest part is excluded, has the lowest Ac, Se, and Sp of 90.32%, 90.72%, and 90.21%, respectively. In addition, the IMU combinations of CET, CA, and a single C also achieved Acs of over 92% (Ses over 90% and Sps over 92%). This further emphasized that the placement of an IMU on the chest is essential in detecting falls. In the group of two placements, combinations that involved the chest achieved fairly high Acs of over 90% and Sps of over 90%, but Ses as low as 86.94%. Similarly, with only one IMU placement, the chest also presents a much higher efficiency than the others.

It is also interesting to note that the combinations of EA and ET, with IMUs placed on the elbows (PJ) and either the ankles or thighs (PT), also have Acs of over 90%, outperforming the rest of the IMU combinations. Moreover, the fall-detection performance based on PJ only (CEW) and PT only (TA) achieved Acs of 91.26% and 89.20%, respectively, indicating that adding IMUs from PT (either the thighs or ankles) can improve the overall fall-detection Ac by at least 2%.

In summary, the results indicated that the IMU combinations of CEWTA, CEWT, and CET had the best performance among all metrics. Moreover, the chest position proves to be the most important placement of the IMU in fall detection for firefighters.

**Figure 14:**
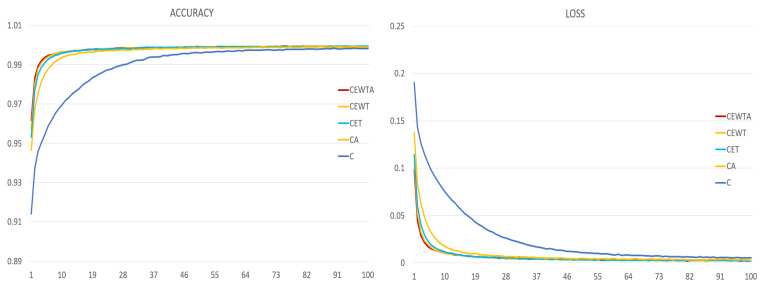
Performance of the five trained models, evaluated with accuracy (**left**) and loss (**right**).

**Figure 15:**
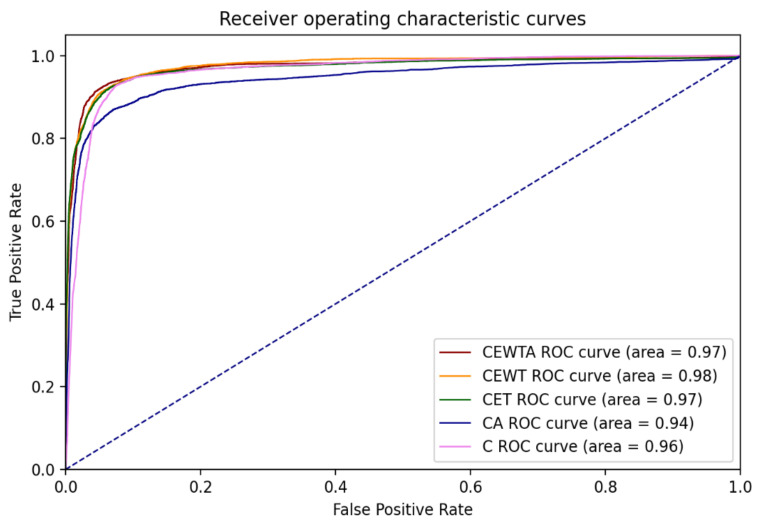
ROC curves and AUC values of the five models.

Furthermore, the most efficient combinations of each quantity group were evaluated. Figure 14 presents the results of the trained models in terms of accuracy and loss. The results illustrate that the fewer the placements, the lower the efficiency in the small-epoch training model, although the accuracy and loss were quite similar after training for 100 epochs. Moreover, Figure 15 illustrates the ROC curves and AUC values of these five models. The results show that all five models perform well and have similar ROC curves. To further evaluate the performance of these models, the efficiencies of each collected activity were compared.

Table 5 presents the detailed performance of these models for each activity. To clarify, the sensitivity values are zero for fall-like activities because no falling happens; hence, no true falling labels appear. According to the average results of these five models, F1 and F6 in the falling activities have lower sensitivities than the other four types. This is mainly because these two activities have an action to reduce the impact before falling, while the others fall directly to the ground. In F1, the knees touch the ground first before falling, and the firefighter sits on the ground, when simulating a backward fall in F2.

Meanwhile, for the fall-like activities, the model is more likely to wrongly predict FL2 (walking with a stoop) as falling. This is because the physical status of the upper body is quite like a fall; hence, it is more difficult to distinguish FL2, if fewer of the lower body’s features are utilized. It is also important to notice that the specificities of the falls are much higher than those of the fall-like activities, which illustrates that fall-like motions indeed have some similarities with falling motions, while normal walking is very different from falling.

In terms of the performance of each model, all five models are sufficient for fall detection (Se over 90% and Ac over 92%); however, the CA and C models are insufficient for a specific fall activity (F2 and F4, respectively). This illustrates that it is difficult to detect some of the falling activities with fewer IMUs and fewer features. Moreover, CEWTA shows a better ability to distinguish falls and non-falls because the specificity of each activity is generally higher than that of the other models. This illustrates that the fall-detection system can indeed be improved by using more IMUs in different positions. In general, the IMU combinations of CEWTA, CEWT, and CET performed the best in each activity.

Van Thanh et al. devoted considerable effort to FDSs for firefighters. Their group presented an acceleration-based algorithm in [27] and proposed four improved algorithms in [28]. The four improved algorithms all have reduced physical performance because of a double-check method used to reduce false detections. Algorithm 1 utilized all features, while the others used parts of the features. Table 6 shows a performance comparison with their studies, as well as some recent FDS studies in other fields.

First, the proposed method only utilized a low data-sampling rate (15 Hz) to achieve higher performance in sensitivity, specificity, and accuracy rate, compared to the other studies (100 Hz), which indicates a cost reduction in the data-processing complexity and power consumption. Compared with the results of the Van Thanh group, the improved algorithms have higher sensitivities than the proposed method, while the one without the double-check algorithm is less efficient. Meanwhile, the algorithm presented by [27] also indicated that fall detection with a single sensor showed worse performance, compared with our multisensory approach.

In comparison with the FDSs in other fields, the results illustrated that IMUs on the ankles [43] and wrists [44] are less efficient than our proposed method in all metrics. However, the study in [42] performed better than our method, which also suggests that a higher sampling rate (100 Hz) could improve the fall-detection accuracy. Moreover, according to [42] and our results, the optimal placement of the IMU could not only be on the chest, but also on the trunk. In general, this proposed study delivers high fall-detection performance by considering the motions from both the upper and lower parts of the body, which increased the detection probability.

## 5. Conclusions

This paper proposed a novel wearable FDS fall-detection for firefighters by embedding motion sensors on the firefighting PPC. The study revealed that the classification of falls and fall-like activities can be distinguished with an accuracy of approximately 94% with all nine IMUs embedded. The study also revealed that an IMU placed on the chest was critical for achieving the best fall-detection performance. Furthermore, the study also concluded that placing IMUs on the chest, elbows, and thighs could also achieve an acceptable fall-detection performance with higher cost efficiency.

In this study, the simulated falling events were based on the experiences and feedback collected from firefighters. This preliminary study denoted the potential of wearable embedded motion sensors for identifying the falling activities of firefighters. Furthermore, the proposed study achieved results similar to those of existing studies, but with a lower sampling rate; hence, reducing the computation cost in general.

A detail plan that covers the ethics and safety issues are currently ongoing and higher realistic falling events will be collected in the future, potentially during actual firefighting rescue missions. Meanwhile, alternative sensors to reduce the false detection will be evaluated. Future studies include fall detection in actual firegrounds, and exploring alternatives to reduce false fall detections, such as utilizing firefighters’ physiological signals (heart rate, brainwave signal) and fireground environmental conditions (CO${}_{2}$ concentration level, etc.). The proposed smart wearable PPC can also be expanded to detect the micro-activities of firefighters, which may increase their safety. In addition, future work also considers the new design of wireless communication framework and infrastructure that can meet the requirements of firefighting activities. Moreover, the sensor components and circuits need further improvements in the aspects of heat-proof, water-proof and washable to overcome the damage issues under the harsh environment in fireground.

## Figures and Tables

**Table 1:** Components and their respective specifications in the sensing module.

Components	Specification
IMU	Triaxial accelerometer
	Triaxial gyroscope
	Triaxial magnetometer
	Operating voltage: 3 V to 5 V
Seeeduino XIAO MCU	Operating voltage: 3.3 V/5 V
	CPU: 40 MHz ARM Cortex-M0+
	Flash memory: 256 KB
	RAM: 32 KB
	Size: 20 × 17.5 × 3.5 mm
	I2C: 1 pair
TCA29548A multiplexer	Operating voltage: 3V to 5V
	I2C: 8 pairs
JDY-18 BLE	Operating voltage: 1.8 V to 3.6 V
	BLE version: 4.2
	Frequency: 2.4 GHz
	Size: 27 × 12.8 × 1.6 mm
Lithium-lon battery	Power supply: 3.7 V
	Capacity: 400 mAh

**Table 2:** Details of activities recorded in the dataset.

Code	Type	Activity	Trials for Each Subject	Total Trials
F1	Falls	forward falls using knees	5	70
F2		forward falls using hands	5	70
F3		inclined falls left	4	56
F4		inclined falls right	4	56
F5		slow forward falls with crouch first	3	42
F6		backward falls	3	42
FL1	Fall-like	crouch	4	56
FL2		walk with stoop	4	56
FL3		sit	3	42

**Table 3:** Codes for the IMU locations.

Placement	Chest	Elbows	Wrists	Thighs	Ankles
**Code**	C	E	W	T	A

**Table 4:** Performance of 30 IMU combinations.

IMU Quantity	Combination	AUC	Se	Sp	Ac
9	CEWTA	0.97	92.25%	94.59%	94.10%
7	CEWT	0.98	91.22%	94.72%	93.98%
7	CEWA	0.95	89.04%	94.25%	93.15%
7	CETA	0.95	88.01%	95.37%	93.82%
7	CWTA	0.95	90.35%	94.21%	93.38%
8	EWTA	0.94	90.72%	90.21%	90.32%
5	CEW	0.94	88.72%	91.94%	91.26%
5	CEA	0.95	88.39%	92.24%	91.43%
5	CWT	0.98	88.39%	92.24%	91.43%
6	EWA	0.93	85.06%	92.42%	90.87%
6	EWT	0.96	89.14%	91.42%	90.94%
5	CWA	0.96	90.84%	93.02%	92.56%
5	CET	0.97	91.61%	94.06%	93.55%
5	ETA	0.96	90.54%	92.30%	91.93%
5	WTA	0.93	90.54%	92.30%	91.93%
3	CE	0.96	92.88%	89.92%	90.54%
3	CW	0.95	90.96%	93.49%	92.96%
3	CT	0.95	86.94%	92.97%	91.70%
3	CA	0.94	90.23%	93.97%	93.18%
4	TA	0.92	83.92%	90.61%	89.20%
4	ET	0.91	85.34%	92.19%	90.75%
4	EA	0.95	87.40%	93.49%	92.21%
4	WT	0.91	85.99%	84.76%	85.02%
4	WA	0.90	81.98%	90.72%	88.88%
4	EW	0.94	83.01%	90.75%	89.14%
2	E	0.91	85.08%	88.70%	87.94%
2	W	0.84	71.99%	80.65%	78.83%
2	T	0.88	78.56%	86.56%	84.87%
2	A	0.89	73.97%	92.44%	88.55%
1	C	0.96	92.82%	92.43%	92.51%

**Table 5:** Detection efficiency for each fall activity.

**Activity**	**CEWTA**			**CEWT**			**CET**		
	**Se**	**Sp**	**Ac**	**Se**	**Sp**	**Ac**	**Se**	**Sp**	**Ac**
F1	91.45%	96.87%	95.42%	87.28%	98.40%	95.42%	89.11%	98.25%	95.80%
F2	94.48%	98.34%	97.15%	93.54%	98.26%	96.81%	95.22%	98.38%	97.41%
F3	96.55%	97.20%	97.00%	95.69%	97.58%	97.07%	97.09%	97.10%	97.10%
F4	95.02%	98.72%	97.62%	94.91%	99.02%	97.79%	95.37%	98.72%	97.72%
F5	96.62%	97.40%	97.22%	98.46%	97.22%	97.50%	97.23%	97.22%	97.22%
F6	78.38%	99.05%	92.80%	80.70%	99.27%	93.66%	76.71%	99.11%	92.33%
FL1	0%	90.31%	90.31%	0%	90.06%	90.06%	0%	89.38%	89.38%
FL2	0%	89.92%	89.92%	0%	84.17%	84.17%	0%	82.21%	82.21%
FL3	0%	87.79%	87.79%	0%	89.87%	89.87%	0%	87.60%	87.60%
Total	92.25%	94.59%	94.10%	91.22%	94.72%	93.98%	91.61%	94.06%	93.55%
**Activity**	**CA**			**C**			**Average**		
	**Se**	**Sp**	**Ac**	**Se**	**Sp**	**Ac**	**Se**	**Sp**	**Ac**
F1	91.25%	94.31%	93.49%	90.84%	95.31%	94.11%	89.99%	96.63%	94.85%
F2	81.84%	95.89%	91.58%	97.66%	95.81%	96.38%	92.55%	97.34%	95.87%
F3	93.64%	97.44%	96.27%	99.68%	95.80%	97.00%	96.53%	97.02%	96.89%
F4	94.61%	98.08%	94.07%	84.14%	96.61%	92.90%	92.81%	98.23%	96.02%
F5	98.46%	96.32%	96.80%	96.61%	97.04%	96.94%	97.48%	97.04%	97.14%
F6	78.38%	97.60%	91.79%	88.55%	97.88%	95.06%	80.54%	98.58%	93.13%
FL1	0%	83.72%	83.72%	0%	99.61%	99.61%	0%	90.62%	90.62%
FL2	0%	86.88%	86.88%	0%	80.88%	80.88%	0%	84.81%	84.81%
FL3	0%	89.99%	89.99%	0%	93.05%	93.05%	0%	89.66%	89.66%
Total	90.23%	93.97%	93.18%	92.82%	92.43%	92.51%	/	/	/

**Table 6:** Comparison of fall-detection results with the proposed FDS and some previously developed FDSs, where SR, Se, Sp, and Acc represent the sampling rate of IMUs, sensitivity, specificity, and accuracy, respectively.

Reference	Application	Methodology	Algorithm	SR	Se	Sp	Ac
Van et al. (2018) [27]	Firefighters	1 3-DOF accelerometer and 1 barometer on the thigh pocket, and 1 CO sensor on the mask (they raised 4 algorithms in [27] and 1 algorithm in [28])	Algorithm 1	100 Hz	100%	100%	100%
			Algorithm 2		100%	94.44%	95.83%
			Algorithm 3		100%	90.74%	93.05%
			Algorithm 4		100%	91.67%	93.75%
Van et al. (2018) [28]			Algorithm 1		88.9%	94.45%	91.67%
Shi et al.(2020) [42]	Elderly	1 IMU on waist	/	100 Hz	95.54%	96.38%	95.96%
AnkFall (2021) [43]		1 IMU on ankle	/	100 Hz	76.8%	92.8%	/
Kiprijanovska et al. (2020) [44]	Ordinary being	2 IMUs in 2 smartwatches	/	100 Hz	90.6%	86.2%	88.9%
Proposed method	Firefighters	9 9-DOF IMUs on the chest, wrists, elbows, thighs and ankles	/	15 Hz	92.25%	94.59%	94.10%

## Data Availability

Not applicable.
